# Applied Improvisation to Improve Mental Health Training

**DOI:** 10.1192/j.eurpsy.2025.1827

**Published:** 2025-08-26

**Authors:** J. D. Fekete, M. Hainselin, K. Eklicsné Lepenye, P. L. Kanizsai, M. Jouin

**Affiliations:** 1 Department of Languages for Biomedical Purposes and Communication, University of Pécs, School of Medicine, Pécs, Hungary; 2 Université de Picardie Jules Verne, Amiens, France; 3 Department of Emergency Medicine, University of Pécs, School of Medicine, Pécs, Hungary

## Abstract

**Introduction:**

Medical improvisation, or HPTI (Health Professional Training Improv), enhances critical skills in healthcare professionals. These skills include communication, empathy, time pressure management, and creative problem-solving. Our presentation aims to showcase a practical application of HPTI, demonstrating its relevance and effectiveness in mental health training.

**Objectives:**

In this presentation, we will demonstrate our training method, including a short session with exercises adapted to healthcare professions. The session is divided into four parts. Each part is designed to maximize engagement and learning, with debriefing sessions to clarify teaching objectives and foster reflective practice.

**Methods:**

Warm-Up Exercises: Physical and vocal exercises to optimize communication and awareness. Improvisation Techniques: Exercises focused on mastering emotions and various forms of communication, allowing participants to explore their strengths and limitations. Medical Scenario Applications: Short clinical scenarios to emphasize the cognitive and affective dimensions of empathy. Review of Existing Workshops and Research: Presentation of current workshops and research to encourage further exploration and application in training. Each part includes a debriefing session to clarify teaching objectives, develop a reflective approach, and identify areas for improvement.

For our ongoing comparative study we have implemented the following questionnaires for our training participants: IUS (uncertainty intolerance scale) and Acceptance and Action Questionnaire-II (AAQII). A total of 50 students participated in our research in both countries.

**Results:**

Hungarian training participants included medical school students in Hungary and fourth-year speech therapy students in France and included professional scenarios based on stress and emotion management, interdisciplinary collaboration and collaboration with the patient, and communication, with debriefing on their performance. The data is currently being collected.

**Conclusions:**

Applied improvisation through HPTI offers a valuable approach to improving mental health training. By enhancing communication, empathy, and problem-solving skills, healthcare professionals can provide better patient care and navigate their roles more effectively. Our presentation demonstrates these techniques and encourages their integration into regular training programs.

**Disclosure of Interest:**

None Declared

